# Development of a complex intervention to support the initiation of advance care planning by general practitioners in patients at risk of deteriorating or dying: a phase 0-1 study

**DOI:** 10.1186/s12904-016-0091-x

**Published:** 2016-02-11

**Authors:** Aline De Vleminck, Dirk Houttekier, Luc Deliens, Robert Vander Stichele, Koen Pardon

**Affiliations:** End-of-Life Care Research group, Ghent University & Vrije Universiteit Brussel (VUB), Laarbeeklaan 103, 1090 Jette, Belgium; Department of Medical Oncology, Ghent University Hospital, Ghent, Belgium; Heymans Institute, Ghent University, Ghent, Belgium

**Keywords:** Advance care planning, General practice, Intervention, Implementation, End-of-life care

## Abstract

**Background:**

Most patients with life-limiting illnesses are treated and cared for over a long period of time in primary care and guidelines suggest that ACP discussions should be initiated in primary care. However, a practical model to implement ACP in general practice is lacking. Therefore, the objective of this study is to develop an intervention to support the initiation of ACP in general practice.

**Methods:**

We conducted a Phase 0-I study according to the Medical Research Council (MRC) Framework. Phase 0 consisted of a systematic literature review about the barriers and facilitators for GPs to engage in ACP, focus groups with GPs were held about their experiences, attitudes and concerns regarding initiating ACP in general practice and a review of ACP interventions to identify potential components for the development of our intervention. In Phase 1, we developed a complex intervention to support the initiation of ACP in general practice in patients at risk of deteriorating or dying, based on the results of Phase 0. The complex intervention and its components were reviewed and refined by two expert panels.

**Results:**

Phase 0 resulted in the identification of the factors inhibiting or enabling GPs’ initiation of ACP and important components underpinning existing ACP interventions. Based on these findings, an intervention was developed in Phase 1 consisting of: (1) a training for GPs in initiating and conducting ACP discussions, (2) a register of patients eligible for ACP discussions, (3) an educational booklet on ACP for patients to prepare the ACP discussions that includes general information on ACP, a section on the role of GPs in the process of ACP and a prompt list, (4) a conversation guide to support GPs in the ACP discussions and (5) a structured documentation template to record the outcomes of discussions.

**Conclusion:**

Taking into account the barriers and facilitators for GPs to initiate ACP as well as the key factors underpinning successful ACP intervention in other health care settings, a complex intervention for general practice was developed, after gaining feedback from two expert panels. The feasibility and acceptability of the intervention will subsequently be tested in a Phase II study.

## Background

Many patients receive inappropriate or futile care at the end of life and this mostly results from a mismatch between the needs of patients and the norms of current practice [1]. A possible response to this concern is advance care planning, as it is a means to discuss patients’ potential needs and care preferences during their illness trajectory. Advance care planning (ACP) is a process of discussions with a patient about their wishes for future healthcare, in preparation for a time when they might lose capacity. ACP is the process by which patients discuss and reflect with their care providers upon topics such as goals and preferences for future care, quality of life, decision-making preferences, fears or anxieties, and also palliative care options, do-not-resuscitate orders, end-of-life decisions and surrogate decision-making in future disease stages [2, 3]. These discussions may or may not result in the documentation of these decisions in an advance directive (AD) and the appointment of a surrogate decision-maker [4].

ACP discussions can play a major role in facilitating adaptation to illness realities by providing patients with information about diagnosis and prognosis, by leading to appropriate decision-making, by alleviating anxiety and by improving quality of life throughout the trajectory of the illness [5, 6]. Previous studies have shown that ACP interventions stimulate discussions about goals of care between patients and their care providers [7, 8], improve concordance between a patient’s preferences and the end-of-life care they receive [9–11] and improved the quality of care at the end of life [12] and they are also associated with positive family outcomes such as improved satisfaction with care and reduced stress and anxiety [9]. Effective ACP discussions support not only end-of-lifecare but quality of life throughout the illness trajectory, including the period before death is imminent [13].

Most patients with serious chronic illnesses are treated and cared for over a long period of time in primary care [14]. Current international guidelines suggest that ACP discussions should be initiated in primary care and that it should be offered to all patients with a chronic life-limiting illness in anticipation of deterioration [4]. Initiating ACP optimally requires a proactive approach by a health care professional who is likely to have a good knowledge of the patient in terms of medical, psychosocial and social background [15]. Given their often longstanding and trusting relationship with patients, including in Belgium, it is assumed that general practitioners (GPs) have good knowledge of the patient and family context. They are also according to other health care professionals [16] in an ideal position to initiate and facilitate timely a structured discussion about the patient’s wishes for future care [1, 17–19].

However, a practical model to implement ACP in general practice is lacking and a cross-national survey showed that only a minority of patients in Belgium had discussed treatment preferences with their GP [20]; GP-patient discussion of treatment preferences occurred for only 25 % of patients in Belgium in the last three months of life. Therefore, our study aims to develop an intervention to support the initiation of ACP in general practice following the UK Medical Research Council’s (MRC) guidance for developing complex interventions [21].

## Methods

### Study design

**Fig. 1:**
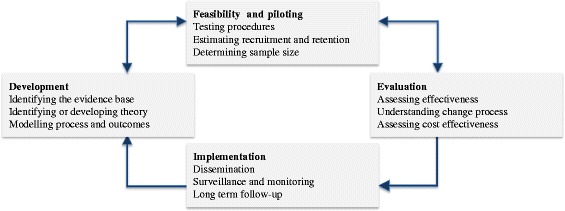
MRC framework for design and evaluation of complex interventions to improve health

The development of this intervention was conducted following the Medical Research Council’s (MRC) framework on complex intervention design [22, 23]. The MRC framework addresses strategies for developing and evaluating complex interventions and proposes a phased approach going from Phase 0 to Phase IV, which take place as an iterative process (Fig. 1) [21]. The phases include exploring relevant theories and potential components for the intervention (Phase 0) and modelling the preliminary complex intervention by selecting the main components of the intervention (Phase I), pilot-testing the preliminary intervention (Phase II), developing the definitive RCT and testing the effectiveness of the intervention (Phase III), and implementing the intervention over the long term (Phase IV). According to the MRC framework, this is a Phase 0-I study comprising the development and modelling of a preliminary complex intervention.

### Phase 0: Exploring potential components of the intervention

Three methods were applied to provide information and evidence for the key components of the intervention. The key components of an intervention refer to the features (i.e. building blocks) of a program that are judged necessary and most effective to produce the desired outcomes [24]. Key components are intended to be, or have been, demonstrated through research to be positively associated with the outcomes that address the identified needs.

First, we performed a systematic literature review about the barriers to and facilitators for GPs to engage in ACP, to identify the relevant factors inhibiting or enabling their initiation of ACP in practice and to guide the choice of intervention components that could overcome the modifiable barriers and enhance the facilitators. Eight qualitative studies and seven cross-sectional studies were included for data-extraction. For more information on the methods of this systematic review, we refer to the published article [25].

Second, focus groups with GPs were held covering their experiences, attitudes and concerns regarding initiating ACP in general practice and investigating their reasons for initiating or not initiating ACP discussions [26]. Five focus groups were held with a purposefully sampled group of GPs to maximize variation in experience, age and practice (*n* = 36). The participants’ characteristics are presented in Table 1. For more information on the different recruitment procedures of these focus groups, we refer to the published article [25]. GPs’ experiences and perceptions were used for further delineating the key components of the intervention that would target the specific barriers and facilitators for GPs in Belgium.

**Table 1:** Characteristics of participating GPs in the focus groups (*N* = 36)

Characteristics	FG 1 (*n* = 9)	FG 2 (*n* = 11)	FG 3 (*n* = 4)	FG 4 (*n* = 5)	FG 5 (*n* = 7)	Total
Sex						
Male	5	7	4	5	6	27
Female	4	4	0	0	1	9
Age (years)						
≤29	1	0	0	0	0	1
30–39	1	2	0	1	1	5
40–49	5	3	1	2	2	13
50–59	1	5	1	1	1	9
60–69	1	1	2	1	3	8
≥70	0	0	0	0	0	0
Practice location						
Urban	9	0	0	0	0	9
(Semi-)Rural	0	11	4	5	7	27
Number of terminal patients in their practice in the last year						
None	2	1	1	0	0	4
1–3	3	3	1	2	1	10
4–6	3	1	2	2	3	11
7–9	0	1	0	0	1	2
≥10	1	5	0	1	2	9
Active in a palliative home care team						
Yes	0	0	0	0	2	2
No	9	11	4	5	5	34
Clinical work experience (years)						
1–9	2	2	0	0	0	4
10–19	2	1	0	2	2	7
20–29	3	4	2	1	2	12
≥30	2	4	2	2	3	13

Third, a rapid review was conducted to identify the key features underpinning successful ACP interventions. A rapid review has a short timeframe, the specified research question may include broad PICO’s, sources may be limited and the data can be summarized descriptively, which fitted the aims of this literature search. We searched in Medline for journal articles with the keywords ‘advance care planning’ and publication type ‘systematic review’. Two recent systematic reviews on ACP interventions were identified [27, 28]. From these systematic reviews, we retrieved the intervention studies with a successful outcome on their intended outcomes. These studies were read in full by the research team and analysed and categorized in an inductive way for their components in order to obtain a comprehensive overview of key features underpinning successful interventions and to identify potential components for the development of our intervention.

### Phase I: Modelling the intervention to general practice

Based on the results of Phase 0, a first draft of a preliminary complex intervention to support the initiation of ACP in general practice was developed by the research team, by selecting appropriate intervention components. Subsequently, the first draft of the complex intervention was presented to expert panels which reviewed the key components and the possible and best course of action to implement the intervention in practice. For the composition of the expert panels, we purposefully sampled either GPs, persons with considerable experience in conducting ACP conversations, academics in the field ACP or patient-physician communication and/or persons experienced in giving communication trainings to physicians. Most participants in the expert panels had experience in a number of these fields. The two expert panels (*n* = 4, *n* = 5) were held in January 2015 and consisted of five GPs, one hospital geriatrician, one palliative care consultant and two academic researchers (psychologists) with expertise in the field of ACP and health care communication. During the panels, the experts were asked to evaluate the completeness of the intervention’s components, to review the components on feasibility and acceptability and to identify the implementation barriers for each component. The expert panels were both consulted in a two-hour long meeting by ADV and KP. The panel discussions were audiotaped (for which the participants gave verbal consent) and transcribed verbatim by ADV. The results were categorized for each component and further analyzed within the research team to refine the intervention.

### Ethical aspects

The research protocol for the qualitative focus group study was approved by the Commission of Medical Ethics of the University Hospital of Brussels. A signed informed consent was obtained from each participant before the focus group interview. Anonymity was assured by removing participant information that could lead to identification from the transcripts.

## Results

### Phase 0: Exploring potential components of the intervention

#### Identification of the factors inhibiting or enabling GP initiation of ACP

Both the systematic review and the focus groups showed that GP, patient and healthcare system factors all influence the initiation of ACP.

1. GP factors influencing the initiation of ACP
  A lack of confidence, skills and knowledge about ACP and how to initiate it were identified as important barriers inhibiting GPs from holding ACP discussions. Many GPs felt poorly prepared to conduct ACP discussions and a lack of awareness of the different components of ACP was shown through their varying conceptualisations of it. The difficulty of defining the right time to initiate ACP was also reported as an important barrier for the GPs. Especially in patients with a less predictable disease course such as dementia or chronic heart failure, they lacked awareness of the key moments to initiate discussions. Difficulties with judging a patient’s mental capacity to participate in ACP and concerns about the legal implications of following their documented wishes were also reported as barriers. Being aware of the potentially positive outcomes of ACP and having positive attitudes towards anticipating future scenarios were identified as important facilitating factors to initiating ACP, as well as positive experiences with ACP in the past.
2. Perceived patient factors influencing the initiation of ACP
  Most GPs in the focus groups considered patients suffering from a life-limiting illness such as cancer to be most eligible for initiating discussions with. However, a barrier that was often mentioned was the concern that initiating ACP discussions too early might deprive patients of hope or create anxiety. Both the patient’s denial or lack of awareness about the prognosis of a serious illness were identified as patient-related factors that contribute to the challenges of GPs initiating ACP. Many GPs also expressed concerns about patients’ lack of understanding regarding ACP. Knowing that a patient is prepared to participate in ACP or the patient initiating an ACP discussion themselves were perceived as important facilitators for GPs to engage in these discussions.
3. Healthcare system factors influencing the initiation of ACP
  Knowing and caring for the patient for a long time was identified as an important facilitating factor for engaging in ACP discussions while a lack of time discourages GPs from initiating them during routine consultations. The lack of a central system for recording the patient’s wishes across different health care settings also contributed to the perceived irrelevance of ACP and was perceived as a challenge to initiating it. Many GPs expressed uncertainty about the usefulness of ACP or ADs as these are not always readily available in a patient’s medical records or consistently recorded across the health care system.

#### Key components underpinning ACP interventions

The examination of ACP interventions identified four common features underpinning successful ACP interventions: 1) the involvement of a trained or experienced facilitator, 2) a selection process to identify patients eligible for ACP, 3) structured and patient-centred ACP discussions and 4) the opportunity to complete ACP documents (Table 2).

**Table 2:** Summary of key features underpinning ACP interventions

*A) Trained or experienced facilitators* In all interventions the ACP discussions were facilitated by a trained health care professional (mostly nurses or allied health workers) [9–11, 43–46] or by health care professionals already experienced in counselling and communicating with patients about ACP, such as social workers or palliative care physicians [7, 47]. The facilitator trainings ranged from half a day to two days and used a competency-based educational approach, comprising interactive discussions about the key components of ACP, role play exercises, reading materials, and learning to assess a person’s capacity to engage in ACP [9–11, 43, 45, 46]. A minority of studies did not provide training to the health care professionals involved [8, 48, 49]. *B) Identification of patients* In most interventions, patient selection was focused on those with a serious advanced life-limiting illness, such as advanced cancer, COPD, end-stage renal disease or end-stage congestive heart failure [7, 8, 10, 43, 44, 47, 50, 51]. Other indicators used to select patients with whom ACP was initiated were age (e.g. all patients ≥65 years) [9, 45, 47, 48, 50, 51], admission to a health care facility (hospital or nursing home) [9, 44–46, 48] and the expectation of serious complications or death within the next year [10, 43, 44]. *C) Tools* A number of interventions used specific tools such as individualized patient-specific questionnaires about the patient’s preferences for discussing ACP [8], a question prompt list about end-of-life care provided to patients before their consultation to stimulate conversations [7], or educational material about ACP mailed to patients in advance [47, 51] to prepare them and to facilitate patient-centred discussions. Structured preference-elicitation and decision aids for ACP to help patients consider their health care options were also implemented as tools during the ACP discussions [9, 10]. *D) Structured discussions* All interventions included structured discussion of the patient’s values, goals and beliefs [9, 44], an assessment of their understanding of their illness [10, 43, 44, 46], discussion of their future treatment preferences [9–11, 43, 44, 46], the assessment of their surrogates’ understanding of their illness and treatment preferences and their role as health care agents [9–11, 43–45, 48] and the opportunity to complete ADs [9, 10, 43, 45–48]. The discussions reportedly lasted between one and one and a half hours [9, 10, 45]. In all studies, the patient was encouraged to include their family. *E) Completion of ACP documents* Most ACP interventions provided the opportunity to complete ACP documents (e.g. documentation of treatment preferences, appointment of a health care proxy, appointment of a surrogate decision-maker) [9–11, 44–51]. A number of studies reported that the completed documents were filed in the patient’s medical records or charts [9, 11, 46]. In two studies conducted in an inpatient setting, extra time was dedicated during the interdisciplinary team meeting after the ACP intervention to discussion of the care wishes of the patient [11, 44].	

### Phase I: Modelling phase

Table 3 indicates how we linked specific barriers and facilitators to the selection of intervention components. This was informed by the features underpinning successful ACP interventions (Table 2). The final selection of intervention components was further informed by what was considered by the research team as relevant for the context in Belgium, was likely to be feasible and could be implemented as a cohesive intervention. The components of the intervention are: 1) a training program for GPs, 2) an register of patients eligible for ACP, 3) an educational booklet for patients about ACP, 4) a conversation guide to support GPs during discussions and 5) a structured documentation template to record the outcomes of the discussions. The components of the intervention were reviewed by two expert panels to refine and improved the intervention.

**Table 3:** Description of the components within a complex intervention to support the initiation of ACP in general practice

Components of a complex intervention to support the initiation of ACP in general practice (evidence based key feature described in Table 2)	
Component 1: A training program for GPs (A)	
Content of the component to overcome the barriers and enhance the facilitators: • Prior to the training, participants will receive reading materials to ensure baseline knowledge on ACP and the relevant law and to limit the time of the training • The training consists of information provision, case studies addressing the identified barriers to initiating ACP, facilitated group discussion and video demonstration of how to conduct ACP discussions and how to incorporate the initiation of ACP into standard consultations, and role play exercises with feedback from an expert instructor to practice the taught skills, which have been shown to be the best strategies to improve communication skills • GPs will learn about the key elements that should be addressed with patients, including exploring prognostic understanding and acceptance of diagnosis and providing patients with information about prognosis to the degree desired by the patient • Instructions are provided for GPs during the training to collect all information regarding the patient’s health status and treatment options, and to contact other health care professionals when necessary for this. • Training will be led by an expert instructor • After the training sessions, GPs will be able to practice their skills at home through e-simulation exercises with fictive patients	Barriers and facilitators that are adressed by the component: ➢ Lack of skills, knowledge about ACP and confidence to discussions ➢ Lack of awareness about the different elements of ACP ➢ Lack of knowledge about decision-making capacity legislation ➢ Recognizing the relevance of ACP ➢ Positive attitudes towards anticipating future scenarios and initiating ACP ➢ Patients’ denial and lack of awareness about prognosis ➢ Lack of adequate communication between GPs and the multiple clinicians involved in the patient’s care
Component 2: Establishing a register of patients eligible for ACP discussions (B)	
Content of the component to overcome the barriers and enhance the facilitators: • Systematic and selective identification of patients at risk of deteriorating or dying will be done using a pragmatic three-step guide of triggers for when to initiate ACP • During the training, GPs will be taught how to identify eligible patients using these triggers and to set up an ACP register, which is a constantly updated list of patients for whom ACP should be initiated at upcoming appointments	Barriers and facilitators that are addressed by the component: ➢ Most GPs in the focus groups considered patients suffering from a life-limiting illness to be most eligible for initiating ACP with ➢ Difficulties with defining a key moment to initiate ACP ➢ Cancer patients are more easily involved in ACP as opposed to non-cancer patients (eg dementia, advanced organ failure, etc.)
Component 3: Educational booklet about ACP for patients (C)	
Content of the component to overcome the barriers and enhance the facilitators: • The goal of an educational booklet is to provide patients with appropriate information on ACP in advance and to prepare them for a patient-centred discussion adapted to their individual information needs • This educational booklet includes a prompt list • Patients are encouraged to reflect on and clarify their wishes together with their relatives through discussion	Barriers and facilitators that are adressed by the component: ➢ Patients’ lack of understanding regarding ACP ➢ Patients are prepared to participate in ACP
Component 4: Patient-centred ACP discussions with the help of a conversation guide (D)	
Content of the component to overcome the barriers and enhance the facilitators: • The conversation guide includes the following topics: understanding of prognosis, information preferences, prognostic information, patient’s previous experiences with ACP, patient goals and quality of life, fears, acceptable function, family involvement and contains examples of questions and communication tips	Barriers and facilitators that are adressed by the component: ➢ Lack of awareness about the different elements of ACP ➢ Varying conceptualisations of ACP among GPs
Component 5: A structured template for documenting the outcomes of the ACP discussions (E)	
Content of the component to overcome the barriers and enhance the facilitators: • A structured template will provide the opportunity for recording patient preferences, values and goals of care • Instructions are provided for GPs during the training to communicate this document to other involved health care professionals (with patient permission). These instructions are also be included on the templates. • Patients are encouraged by the GP to make this document available for other care providers	Barriers and facilitators that are adressed by the component: ➢ Difficulties in sharing information across the health care system when patients are cared for by (multiple) specialists ➢ Uncertainty regarding the transferability of ACP information as there is no consistent standard for location

1. The training session is based on a two hour educational programme about ACP specifically developed for GPs that has been shown to improve their confidence and ability to undertake ACP conversations with patients [29]. It includes active, practice-oriented strategies such as role-play exercises, feedback, the use of video role modelling, group discussions and feedback during the session as these are educational strategies which have shown to be most effective in improving communication skills [30]. Pre-reading material and information provision will also be a part of the training programme as lack of knowledge about the potentially positive outcomes of ACP and about its legal implications were identified as important barriers. A previous study also showed that improved clinician knowledge about decision-making capacity legislation positively correlated with ACP participation [31]. The experts perceived two hours as too short to complete all training elements and recommended spreading the training over two sessions which would permit reinforcement and allow for home work exercises in between.
2. Standardised triggers for the timely identification of all patients who are at risk of deteriorating or dying in the near future was considered essential to support GPs in defining a key moment and prompting them to initiate ACP discussions. While the timing of ACP must be sensitive to patients’ readiness to enter into such conversations, a pragmatic three-step guide that has been proposed for physicians is to consider ACP if: (a) No is the answer to the surprise question “Would you be surprised if this patient died within the next year?”, (b) the patient’s general health is poor (eg limitations in self-care or multiple hospitalisations), and (c) if disease-specific indicators indicate a poor prognosis (eg advanced organ failure, dementia, progressive malignancies) [1]. Identified eligible patients will be documented in a register. These patients should be invited to consider ACP.
3. To overcome the patient-related barriers to GPs in initiating ACP, an educational booklet for assisting patients and their caregivers and improving their knowledge about ACP was considered helpful to increase patient and family engagement This booklet was based on an already existing booklet ‘Planning your future care’ developed by the University of Nottingham and published by the NHS as part of the Dying Matters campaign in the UK. This booklet was deemed suitable by the expert panels as it includes general information on ACP and a section on the role of GPs in the process of ACP. However, most experts considered this booklet as too long and suggested to shorten it and slightly adapt its content to our target group of patients. A prompt list was also included as research showed that a prompt list helped patients to ask questions about prognosis and end-of-life care, and discuss more issues covered by the question prompt list with their physician [7]. The educational booklet would be given to patients during a routine visit and would facilitate subsequent patient-centred ACP discussions.
4. A conversation guide was developed to support GPs and to assure a better uptake of all the key elements of ACP discussions [13]. A draft was made based of a conversation guide for communication about serious illness care goals that was developed on the basis of a review and synthesis of best practice and afterwards validated in the expert panels. The key elements addressed in the conversation guide are: 1) understanding of prognosis, 2) decision making and information preferences, 3) prognostic disclosure, 4) patient goals and quality of life, 5) fears, 6) level of functioning acceptable to the patient, 7) trade-offs that might be necessary to achieve different outcomes and 8) family involvement and choosing an appropriate surrogate decision-maker. The experts suggested shortening the conversation guide to a one-page topic list. Most experts found a structured conversation guide useful to assure the completion of key steps in the conversations, but emphasized that it should be made clear in the training sessions that the conversation guide should not be used as a checklist or static script.
5. A documentation template was developed that is standardised, simple and patient-friendly and that allows the opportunity to record and document the outcomes of ACP conversations. In the expert panels consensus was reached that the document should be complementary to an AD and record key information such as the patient’s values and goals, quality of life, fears and anxieties, etc. to help guide complex decisions. It was decided that this template could follow the same structure as the conversation guide. To improve the exchange of information about patient values and goals, instructions are also provided for GPs to communicate this document to other involved health care professionals with the patient’s permission, and patients will be encouraged by the GP to make this document available to other care providers.

## Discussion

This article describes in detail the development process of an intervention to support the initiation of ACP in general practice consisting of five key components: 1) a training program for GPs in initiating and conducting ACP, 2) a register of patients who should be invited for ACP, 3) an educational booklet for patients to prepare them for ACP discussions, 4) a conversation guide to support GPs in ACP discussions and 5) a structured documentation template to record the discussions.

An important strength of the study is that it was systematically developed using the MRC framework for the development and evaluation of complex interventions. To our knowledge, this is the first development of a complex intervention aiming to support the initiation of ACP for patients at risk of deteriorating or dying in general practice. It has been recognized that the development of interventions calls for a systematic approach with a strong rationale for design and for the explicit reporting of the development process [32]. Since its publication, a number of researchers in palliative care have applied the MRC framework to develop their interventions [33–35] and we may affirm that this Framework definitely has potential as a broad guide to help researchers develop a complex intervention. The MRC Framework guidance suggests the use of appropriate quantitative and/or qualitative methodologies depending on the specific objectives of the phases as well as a specific study design taking into account the theoretical basis, any evidence on the issue and the context’s specificity. However, we experienced some shortcomings within the Framework’s guidance. For example, we did not find any details or references in the guidance on how a complex intervention could be developed and/or modeled starting from interventions, which are mostly practice-based and not often rely on theory, as is the case in the field of ACP, or on how to identify the core components as part of the intervention.

Nonetheless, by using different research methods, data-triangulation was achieved, which we believe is an important strength of our Phase 0-I study. Moreover, the expert panel helped to identify the possible and best course of action to implement the intervention in practice. However, some limitations of our Phase 0-I study have to be acknowledged. Firstly, our search strategy was limited to systematic reviews about the effect of ACP interventions, which were screened for successful intervention studies that were then analysed by the research team for their key components. Observational studies as well as studies in which ACP was one component of a larger intervention were not included in our scope. Including different study designs such qualitative studies could have provided additional insights. Second, the focus group composition may have presented a limitation. Most of the participating GPs were male (*n* = 27), so female GPs (*n* = 9) were underrepresented, as were GPs younger than 39 years (*n* = 6 vs. *n* = 30). Third, the composition of the expert panels might have presented a limitation. Although the panels were purposively sampled, not all relevant disciplines were represented such as patient representatives. Nonetheless, academic researchers and experts in the field of health care communication and the development of booklets and decision aids were attending. Third, a key component mentioned in a recently published clinical review on ACP could not be included in the intervention. It was found that not only a structured template for documenting the discussions should be provided, but also that a specific page in the electronic medical record (EMR) should be designated that can be easily accessed by all health care professionals involved [13]. A small study recently examined the quality of ACP documentation templates in the EMR [36]. This study found a great potential for making ACP documentation standardized and easily accessible in EMRs. The vast majority of notes in patients’ EMR contained important information regarding desired surrogate decision-makers and care preferences. In Belgium, cross-setting electronic health records do not exist yet and can thus not be used as a tool for communication across the health care system. Therefore, GPs are given the instruction to notify all involved health care professionals by communicating the documentation template that records the outcomes of the ACP discussions (with patients’ approval).

The key components of our intervention largely correspond with the key factors identified in a recent narrative review on the enhancement of patient-professional communication about end-of-life issues. This narrative review did include intervention studies as well as observational studies and qualitative studies [37]. Important features of a successful ACP model are shown to be focused participant-led training, the use of effective communication to improve patient understanding and flexible patient-led ACP discussions. The authors argue that the development of an ACP intervention for patients with life-limiting conditions should include careful consideration of these features. Findings also showed that ACP should ideally take place over a number of meetings, with a trained professional with sufficient time to answer questions. ACP discussions should focus more on the goals of care than specific treatments and discussions should be tailored to the individual patient. Lastly, discussions should be supported with written documentation. A number of recently published systematic reviews on the effects of ACP on end-of-life care confirmed that complex ACP interventions may be more effective in meeting patients’ preferences than written documents alone [27, 28]. Interventions focusing on ADs as well as those that also included communication about end-of-life care increased the chances of an AD being completed and the occurrence of end-of-life care discussions between patients and healthcare professionals. But interventions that also included communication about ACP improved concordance between preferences for care and delivered care [28].

The developed ACP intervention differs from other interventions, most of which tend to be delivered by a trained facilitator who is not always knowledgeable about the clinical profile and social context of individual patients. This study took as the point of departure the proposition that ACP discussions should be initiated and facilitated by a health care professional with whom the patient feels comfortable about discussing their wishes and goals of care. The evidence suggests that other health care professionals can successfully conduct ACP, yet patients continue to state that they expect their primary care physician to initiate such conversations [38]. In the Belgian health care system GPs are core providers of primary care and the majority of people have a GP with whom they have often built up a long-term relationship. GPs are usually cognisant of both the physical and non-physical domains of the patient’s health. So by targeting GPs, the time consuming nature of ACP conversations can possibly be limited (in contrast to the reported discussions of 1.5 h in Table 2) because GPs in Belgium mostly know and care for their patients and their families over a long period time.

A study on the implementation and embedding of ACP interventions in routine clinical practice showed that the factors that promote implementation in practice are largely concerned with structural mechanisms [39]. These include prescheduled interventions and administrative procedures applied to selected patients, dedicated teams to organize the interventions, specifically trained facilitators to deliver the interventions and a dedicated document and organisational policies or guidelines to support the process. This intervention takes into account these factors by training GPs to manage the interactional processes with their patients, equipping them with simple tools such as a register for patients eligible for ACP discussions, a conversation guide, and documentation templates to record the ACP discussions. A patient brochure that will assist and increase the knowledge of patients and their caregivers of the potentially positive outcomes of ACP will also increase their willingness to engage in it with their GP. Educating patients on ACP and letting them prepare for the discussions with the educational booklet can also possibly contribute to limiting the time involved in conducting ACP discussions. It is however crucial that such tools are not considered as a replacement for meaningful communication between the GP, their patients and their families; they should rather precede, facilitate and support ACP discussions [40].

Further research should now focus on testing the feasibility and acceptability of the intervention’s components and on explaining how the various components work together in a Phase II study [41, 42]. This is necessary to understand the best way to implement the components in standard care, the mechanisms underlying the complex intervention, to explain why the intervention works or not and to revise the results of Phase 0-I accordingly [23], which will greatly improve the intervention design and evaluation. Having developed and modelled this intervention to support the initiation of ACP in general practice, it will be important to evaluate its effectiveness thoroughly.

## Conclusion

Performing a phase 0-I study according to the MRC framework helped us to develop a complex intervention to support the initiation of ACP in general practice for patients at risk of deteriorating or dying. Taking into account the barriers to and facilitators for GPs to initiate ACP as well as the key factors underpinning successful ACP intervention in other health care settings, we developed and modelled a complex intervention for general practice, after gaining feedback from two expert panels. The develop intervention consists of five key components: 1) a training program for GPs in initiating and conducting ACP, 2) a register of patients who should be invited for ACP, 3) an educational booklet for patients to prepare them for ACP discussions, 4) a conversation guide to support GPs in ACP discussions and 5) a structured documentation template to record the discussions. Further research should now focus on testing the feasibility and acceptability of the intervention’s components and on explaining how the various components work together in a Phase II study.

## Abbreviations

ACP: Advance Care Planning
AD: Advance Directive
GP: General Practitioner
MRC: Medical Research Council
